# Chronic Disease Indicators: 2022–2024 Refresh and Modernization of the Web Tool

**DOI:** 10.5888/pcd21.240109

**Published:** 2024-06-20

**Authors:** Kathleen B. Watson, Susan A. Carlson, Hua Lu, Karen G. Wooten, Magdalena M. Pankowska, Kurt J. Greenlund

**Affiliations:** 1 Division of Population Health, National Center for Chronic Disease Prevention and Health Promotion, Centers for Disease Control and Prevention, Atlanta, Georgia; 2 DB Consulting Group, Bethesda, Maryland; 3 Oak Ridge Institute for Science and Education, Oak Ridge, Tennessee

## Abstract

Easy access and display of state-level estimates of the prevalence of chronic diseases and their risk factors can guide evidence-based decision-making, policy development, and tailored efforts to improve population health outcomes; however, these estimates are often presented across multiple websites and reports. The Chronic Disease Indicators (CDI) web tool (www.cdc.gov/cdi) disseminates state-level data compiled from various data sources, including surveys, vital records, and administrative data, and applies standardized definitions to estimate and track a wide range of key indicators of chronic diseases and their risk factors. In 2022–2024, the indicators were refreshed to include 113 measures across 21 topic areas, and the web tool was modernized to enhance its key features and functionalities, including standardized indicator definitions; interactive charts, graphs, and maps that present data in a visually appealing format; an easy-to-use web-based interface for users to query and extract the data they need; and state comparison reports to identify geographic variations in disease and risk factor prevalence. National and state-level estimates are provided for the overall population and, where applicable, by sex, race and ethnicity, and age. We review the history of CDIs, describe the 2022–2024 refresh process, and explore the interactive features of the CDI web tool with the goal of demonstrating how practitioners, policymakers, and other users can easily examine and track a wide range of key indicators of chronic diseases and their risk factors to support state-level public health action.

**Summary**

**What is already known on this topic?**

The Chronic Disease Indicators (CDI) web tool (www.cdc.gov/cdi) disseminates state-level data compiled from various sources, including surveys, vital records, and administrative data, and applies standardized definitions to estimate key indicators of chronic diseases.

**What is added by this report?**

A refreshed CDI web tool was released in 2024 with 113 indicators and enhancements to key functions, including standardized indicator definitions; interactive charts, graphs, and maps; an easy-to-use web-based data portal; and state comparison reports.

**What are the implications for public health practice?**

The CDI web tool provides practitioners, policymakers, and other users an easy way to examine and track a wide range of key indicators of chronic diseases and their risk factors to support state-level public health action.

## Introduction

In 2018, more than half of US adults had 1 or more of the following 10 chronic conditions: arthritis, cancer, chronic obstructive pulmonary disease, coronary heart disease, current asthma, diabetes, hepatitis, hypertension, stroke, and weak or failing kidneys (1). These and other chronic diseases are the leading causes of illness, disability, and death and the leading drivers of health care costs in the US (2). Several health risk behaviors (eg, smoking, alcohol use, physical inactivity) and social determinants of health affect the prevalence, management, and treatment of chronic diseases (2). Increases in knowledge about risk factors and conditions and recognition of social factors affecting health outcomes have led to an expanded view of the role of public health beyond viewing chronic conditions alone.

State-level estimates of chronic diseases and their risk factors can play an important role in the development of state-level policies, practices, and programs; identification of state-level priority conditions and health risk behaviors; resource prioritization and allocation; and tracking changes over time to assess progress toward state health objectives and the effect of state efforts. In the US, several surveillance systems monitor chronic diseases and their risk factors and can provide state-level estimates (eg, the Behavioral Risk Factor Surveillance System [BRFSS] [3], the Youth Risk Behavior Surveillance System [YRBSS] [4]). Data stewards of systems house the data on their sites and often provide summary estimates via reports or applications. In addition, many programs of the Centers for Disease Control and Prevention (CDC) present estimates of the prevalence or incidence of chronic diseases or their risk factors (5–7). However, users interested in estimates across multiple data sources and different chronic diseases and their risk factors may have difficulty in efficiently extracting data across platforms.

The Chronic Disease Indicators (CDI) web tool (www.cdc.gov/cdi) is designed to provide public health professionals, researchers, and policymakers with valuable data for the surveillance of chronic diseases and their risk factors at the national and state level. The web tool compiles data from various data sources, including surveys, vital records, and administrative data, and applies standardized definitions to estimate and track key indicators. CDI provides estimates from different data sources in the same data set to connect data across the health spectrum, from risk factors and behaviors to treatment and health care outcomes to mortality. CDI is one of the most popular sites for chronic disease data and is in the top 4% (5 of 135) of most-accessed datasets among all of CDC’s chronic diseases datasets. The roots of CDI began in 1999 when a set of surveillance indicators were released by CDC, the Council of State and Territorial Epidemiologists (CSTE), and the Association of State and Territorial Chronic Disease Program Directors (ASTCDPD) (8). During the past 20 years, the indicators have changed, as has the web tool. To better serve users and to improve the efficiency of CDI, a refresh process was initiated in 2022. This process included a re-examination of the indicators and modernization of and enhancements to the web tool.

## History of Chronic Disease Indicators

The original set of 73 indicators was released in 1999 by CDC, CSTE, and ASTCDPD. In 2002, the indicators were revised and expanded to 92 (8). CDC, CSTE, and ASTCDPD collaborated on the 2002 revision. Indicators were evaluated for their relevance to chronic disease prevention and control at the state level and for the availability of data on an annual or biannual basis at the state level for most states. In addition, an attempt was made to select indicators consistent with Healthy People 2010 objectives (9).

In 2012–2013, the indicators were revised with the release of 201 measurable indicators (10). CDC, CSTE, and the National Association of Chronic Disease Directors (NACDD) collaborated to conduct a series of reviews informed by subject-matter expert opinion to make recommendations for revising the indicators. Each recommendation was expected to follow 3 principles: 1) allow states, territories, and large metropolitan areas to uniformly define, collect, and report chronic disease data related to diseases and conditions with a substantial public health effect; 2) ensure that data are consistent with Healthy People 2020 objectives (if possible) (11); and 3) ensure that data are available at the state level for most states and preferably for territories and large metropolitan areas as well.

In 2015, the CDI web tool was released with the 203 indicators published in that year and a few revisions. Revisions included division of the “amount of alcohol excise tax by beverage type” indicator into 3 indicators (beer, wine, distilled spirits), addition of 4 age-specific indicators, and 5 removals due to lack of state-level data.

## 2022–2024 Indicator Refresh

In 2022, CDC initiated a CDI refresh process that began with a re-examination of the indicators. Much like the reduction in the number of objectives for Healthy People 2030, the CDI also underwent a process including a reduction in the number of indicators. With fewer indicators, the CDI increases its focus on the most pressing public health issues, making it easier to find indicators relevant to the public health work being done. CDC programs reviewed the 203 measurable indicators and recommended which indicators to retain, modify, or remove, and proposed new indicators. Recommendations were expected to meet 3 criteria: 1) the disease or condition has a substantial effect on public health; 2) data for the disease or condition are currently available for most states; and 3) the indicator related to the disease or condition is consistent with public health goals at the national (12) or state level. During the refresh process, programs also aimed to ensure that CDI’s presentation of indicators effectively complemented web tools hosted by other CDC programs while retaining its distinctive role as a comprehensive source of data on chronic disease indicators. Funding restrictions prohibited an external process, which was used previously. However, feedback on the refresh process and indicators was obtained from state partners through consultation between CDC programs and relevant partners, as well as presentations and discussions with state-focused groups and organizations, including CSTE, NACDD, and the Association of State and Territorial Health Officials.

The result was a refreshed set of 113 indicators with estimates from 14 data sources (Table 1). Of the 113 indicators, 63 were retained from the previous version, 20 were modified (2 changed data sources and 18 changed measure definitions), and 30 were new (Table 2). Indicators were reorganized into 21 topic areas with the addition of 4 new topic areas in which public health importance and focus has increased during the last several years (Cognitive Health and Caregiving, Health Status, Sleep, and Social Determinants of Health) (Table 2). Indicators from the previous Overarching Conditions topic area were re-organized into other topic areas (n = 9 indicators) or removed (n = 7 indicators). Several indicators are also listed in additional topic areas (Student Health [n = 16 indicators], Older Adults [n = 6 indicators], and Maternal Health [n = 11 indicators]).

**Table 1:** Data Sources for Indicators Included in the 2022–2024 Chronic Disease Indicators Refresh and Modernization

Name	Abbreviation	Link
American Community Survey	ACS	https://www.census.gov/programs-surveys/acs/
Alcohol Epidemiologic Data System	AEDS	https://www.niaaa.nih.gov/publications/surveillance-reports
American Nonsmokers’ Rights Foundation	ANRF	https://no-smoke.org/materials-services/lists-maps/#1518200878061-ebc83fdc-2d6c
Behavioral Risk Factor Surveillance System	BRFSS	http://www.cdc.gov/brfss
Centers for Medicare & Medicaid Services Part A Claims Data	CMS Part A Claims Data	https://www.cms.gov/data-research
Current Population Survey Food Security Supplement	CPS FSS	https://www.census.gov/data/datasets/time-series/demo/cps/cps-supp_cps-repwgt/cps-food-security.html
National Immunization Surveys	NIS	https://www.cdc.gov/vaccines/imz-managers/nis/about.html
National Survey of Children’s Health	NSCH	https://www.census.gov/programs-surveys/nsch.html
National Vital Statistics System	NVSS	http://www.cdc.gov/nchs/nvss
Pregnancy Risk Assessment Monitoring System	PRAMS	https://www.cdc.gov/prams
United States Renal Data System	USRDS	https://www.niddk.nih.gov/about-niddk/strategic-plans-reports/usrds
US Cancer Statistics Data Visualizations Tool	US Cancer DVT	https://www.cdc.gov/cancer/uscs/dataviz/index.htm
Women, Infants, and Children Participant and Program Characteristics	WIC-PC	https://www.fns.usda.gov/pd/wic-program
Youth Risk Behavior Surveillance System	YRBSS	http://www.cdc.gov/YRBSS

**Table 2:** Indicators Included in the 2022–2024 Chronic Disease Indicators Refresh and Modernization, by Topic Area

Indicator	Data source	Compared with 2012–2013 CDI web tool
**Alcohol**		
Alcohol use among high school students^a^	YRBSS	Retained
Binge drinking prevalence among high school student^a^	YRBSS	Retained
Binge drinking frequency among adults	BRFSS	Modified^b^
Binge drinking intensity among adults who binge drink	BRFSS	Modified^b^
Binge drinking prevalence among adults	BRFSS	Retained
Chronic liver disease mortality among all persons, underlying cause	NVSS	Retained
Alcohol use during pregnancy among women with a recent live birth^c^	PRAMS	Modified^d^
Binge drinking during pregnancy among women with a recent live birth who use alcohol^c^	PRAMS	New
Per capita alcohol consumption among persons aged ≥14 years	AEDS	Retained
**Arthritis**		
Arthritis among adults	BRFSS	Retained
Activity limitation due to arthritis among adults with arthritis	BRFSS	Retained
No leisure-time physical activity among adults with arthritis	BRFSS	Retained
Severe joint pain due to arthritis among adults with arthritis	BRFSS	Retained
Work limitation due to arthritis among adults aged 18–64 years with arthritis	BRFSS	Retained
Received health care provider counseling for physical activity among adults with arthritis	BRFSS	New
Have taken a class to learn how to manage arthritis symptoms among adults with arthritis	BRFSS	Retained
**Asthma**		
Current asthma among adults	BRFSS	Retained
Asthma mortality among all person, underlying cause	NVSS	Retained
**Cancer**		
Colorectal cancer screening among adults aged 45–74 years	BRFSS	Modified^e^^,^ f
Cervical cancer screening among women aged 21–65 years	BRFSS	Modified^f^
Mammography use among women aged 50–74 years	BRFSS	Retained
Invasive cancer (all sites combined), incidence	US Cancer DVT	Retained
Colon and rectum (colorectal) cancer mortality among all persons, underlying cause	US Cancer DVT	Retained
Breast cancer mortality among all females, underlying cause	US Cancer DVT	Retained
Cervical cancer mortality among all females, underlying cause	US Cancer DVT	Retained
Lung and bronchial cancer mortality among all persons, underlying cause	US Cancer DVT	Retained
Prostate cancer mortality among all males, underlying cause	US Cancer DVT	Retained
Invasive cancer (all sites combined) mortality among all persons, underlying cause	US Cancer DVT	Retained
**Cardiovascular disease**		
Cerebrovascular disease (stroke) mortality among all persons, underlying cause	NVSS	Modified^e^
Coronary heart disease mortality among all persons, underlying cause	NVSS	Modified^e^
Diseases of the heart mortality among all persons, underlying cause	NVSS	Modified^e^
Taking medicine for high cholesterol among adults	BRFSS	New
High blood pressure among adults	BRFSS	Retained
High cholesterol among adults who have been screened	BRFSS	Retained
Taking medicine to control high blood pressure among adults with high blood pressure	BRFSS	Retained
Hospitalization for heart failure as principal diagnosis, Medicare-beneficiaries aged ≥65 years^g^	CMS Part A claims data	Retained
Pregnancy-related hypertension among women with a recent live birth^c^	PRAMS	New
**Chronic kidney disease**		
Incidence of treated end-stage renal disease	USRDS	Retained
**Chronic obstructive pulmonary disease**		
Chronic obstructive pulmonary disease among adults	BRFSS	Retained
Current smoking among adults with chronic obstructive pulmonary disease	BRFSS	Retained
Hospitalization for chronic obstructive pulmonary disease as any diagnosis, Medicare-beneficiaries aged ≥65 years^g^	CMS Part A claims data	Retained
Hospitalization for chronic obstructive pulmonary disease as principal diagnosis, Medicare-beneficiaries aged ≥65 years^g^	CMS Part A claims data	Retained
Chronic obstructive pulmonary mortality among adults aged ≥45 years, underlying cause	NVSS	Retained
Chronic obstructive pulmonary mortality among adults aged ≥45 years, underlying or contributing cause	NVSS	Retained
**Cognitive health and caregiving**		
Provided care for someone with dementia or other cognitive impairment in the past month among adults	BRFSS	New
Provided care for a friend or family member in the past month among adults	BRFSS	New
Subjective cognitive decline among adults aged ≥45 years	BRFSS	New
Discussed symptoms of subjective decline with a health care professional among adults aged ≥45 years with subjective cognitive decline	BRFSS	New
**Diabetes**		
Diabetes among adults	BRFSS	Retained
Diabetes mortality among all persons, underlying or contributing cause	NVSS	Retained
Diabetes ketoacidosis mortality among all persons, underlying or contributing cause	NVSS	Retained
Gestational diabetes among women with a recent live birth^c^	PRAMS	Retained
**Disability**		
Adults with any disability	BRFSS	Modified^h^
**Health status**		
≥2 Chronic conditions among adults	BRFSS	Modified^e^^,^ f ^,^ h
Frequent physical distress among adults	BRFSS	New
Fair or poor self-rated health status among adults	BRFSS	Retained
Recent activity limitation among adults	BRFSS	Retained
Average recent physically unhealthy days among adults	BRFSS	Retained
Life expectancy at birth	NVSS	Retained
**Immunization**		
Influenza vaccination among adults	BRFSS	New
Influenza vaccination among adults who are at increased risk	BRFSS	Retained
Pneumococcal vaccination among adults aged 18–64 years who are at increased risk	BRFSS	New
Pneumococcal vaccination among adults aged ≥65 years^g^	BRFSS	New
**Maternal health^i^**		
Postpartum checkup among women with a recent live birth	PRAMS	Retained
**Mental health**		
Current poor mental health among high school students^a^	YRBSS	New
Depression among adults	BRFSS	New
Frequent mental distress among adults	BRFSS	New
Average mentally unhealthy days among adults	BRFSS	Retained
Formal postpartum mental health screening among women with a recent live birth^c^	PRAMS	New
Postpartum depressive symptoms among women with a recent live birth^c^	PRAMS	Retained
**Nutrition, physical activity, and weight status**		
Consumed fruit <1 time daily among high school students^a^	YRBSS	Modified^b^
Consumed vegetables <1 time daily among high school students^a^	YRBSS	Modified^b^
Consumed fruit <1 time daily among adults	BRFSS	Modified^b^
Consumed vegetables <1 time daily among adults	BRFSS	Modified^b^
Infants who were exclusively breastfed through 6 months	NIS	Modified^b^
Infants who were breastfed at 12 months	NIS	New
Children and adolescents aged 6–13 years meeting aerobic physical activity guidelines^a^	NSCH	New
Met aerobic physical activity guidelines among high school students^a^	YRBS	Retained
Met aerobic physical activity guidelines for substantial health benefits, adults	BRFSS	Retained
No leisure-time physical activity among adults	BRFSS	Retained
Obesity among WIC children aged 2–4 years	WIC-PC	New
Obesity among adults	BRFSS	Retained
Obesity among high school students^a^	YRBSS	Retained
Consumed regular soda ≥1 time daily among high school students^a^	YRBSS	Retained
**Older adults^j^**		
**Oral health**		
Visited dentist or other oral health care provider in the past 12 months among children and adolescents aged 1–17 years^a^	NSCH	Retained
Receipt of evidence-based preventive dental services in the past 12 months among children and adolescents aged 1–17 years^a^	NSCH	Modified^b^
Visited dentist or dental clinic in the past year among adults	BRFSS	Retained
No teeth lost among adults aged 18–64 years	BRFSS	Retained
≥6 Teeth lost among adults aged ≥65 years^g^	BRFSS	Retained
All teeth lost among adults aged ≥65 years^g^	BRFSS	Retained
Preventive dental care in the 12 months before pregnancy among women with a recent live birth^c^	PRAMS	Retained
**Sleep**		
Short sleep duration among children aged 4 months to 14 years^a^	NSCH	New
Short sleep duration among high school students^a^	YRBSS	New
Short sleep duration among adults	BRFSS	Modified^b^
**Social determinants of health**		
No broadband internet subscription among households	ACS	New
Unemployment rate among persons aged ≥16 years in the labor force	ACS	New
High school completion among adults aged 18–24 years	ACS	Retained
Living below 150% of the poverty threshold among all persons	ACS	Modified^b^
Routine checkup within the past year among adults	BRFSS	New
Lack of social and emotional support among adults	BRFSS	New
Lack of reliable transportation among adults	BRFSS	New
Unable to pay mortgage, rent, or utility bills in the past 12 months among adults	BRFSS	New
Lack of health insurance among adults aged 18–64 years	BRFSS	Retained
Food insecure in the past 12 months among households	CPS FSS	New
Health insurance coverage after pregnancy among women with a recent live birth^c^	PRAMS	New
Health insurance coverage in the month before pregnancy among women with a recent live birth^c^	PRAMS	Retained
**Student health^j^**		
**Tobacco**		
Current use of any tobacco product among high school students^a^	YRBSS	Modified^b^
Current electronic vapor product use among high school students^a^	YRBSS	New
Current smokeless tobacco use among high school students^a^	YRBSS	Retained
Current cigarette smoking among adults	BRFSS	Retained
Quit attempts in the past year among adult current smokers	BRFSS	Retained
Cigarette smoking during pregnancy among women with a recent live birth^c^	PRAMS	Modified^d^
Proportion of the population protected by a comprehensive smoke-free policy prohibiting smoking in all indoor areas of workplaces and public places, including restaurants and bars	ANRF	Retained

Abbreviations: ACS, American Community Survey; AEDS, Alcohol Epidemiologic Data System; ANRF, American Nonsmokers’ Rights Foundation; BRFSS, Behavioral Risk Factor Surveillance System; CMS, Centers for Medicare & Medicaid Services; CPS-FSS, Current Population Survey Food Security Supplement; NIS, National Immunization Surveys; NSCH, National Survey of Children's Health; NVSS, National Vital Statistics System; PRAMS, Pregnancy Risk Assessment Monitoring System; USRDS, United States Renal Data System; US Cancer DVT, US Cancer Statistics Data Visualizations Tool; WIC, Special Supplemental Nutrition Program for Women, Infants, and Children; WIC-PC, Women, Infants, and Children Participant and Program Characteristics; YRBSS, Youth Risk Behavior Surveillance System.

a Indicator is also in Student Health topic area.

b Modified indicators due to changes in measure type.

c Indicator is also in Maternal Health topic area.

d Modified indicators due to changes in case definition.

e Modified indicators due to changes in population.

f Modified indicators due to changes in numerator and denominator.

g Indicator is also in Older Adults topic area.

h Modified indicators due to changes in data source.

i Additional indicators are listed in other topic areas.

j All indicators are listed in other topic areas.

The number of indicators included in the refreshed CDI is a decrease from the previous web tool. With fewer indicators, the CDI web tool can minimize overlap with other tools while still presenting key indicators across multiple chronic diseases and their risk factors, including estimates stratified by sex, race and ethnicity, and age. With this refresh, 120 indicators included in the previous version were removed based on the criteria provided above. Twenty-six indicators were removed because data for the indicator were no longer available for most states. Characteristics of other removed indicators were similar across topic areas and included indicators limited to adults with a condition (eg, prevalence of co-occurring conditions [n = 10 indicators] or immunizations [n = 16 indicators]), indicators limited to women aged 18 to 44 years (n = 16 indicators), indicators related to a support or barrier for a disease or risk behavior (n = 24 indicators), cancer indicators related to site-specific incidence or lower mortality rates (n = 9 indicators), indicators that overlapped with another indicator (n = 13 indicators), and indicators for other characteristics (n = 6 indicators).

Many indicators removed are still available on other topic websites or on data custodian websites. For example, the BRFSS Web Enabled Analysis Tool (WEAT) (https://nccd.cdc.gov/weat/#) has a cross-tabulation application that allows users to produce tables showing numbers and percentages of 1 variable by 1 or more additional variables. WEAT can generate estimates of indicators specific to women aged 18 to 44 years (create crosstabulation tables by sex and age group) and for those with a chronic disease (create crosstabulation tables by presence of a condition [eg, arthritis, diabetes]) to obtain subgroup estimates. Links to topic-specific websites are provided as part of the topic-specific landing pages included with the refreshed CDI web tool and links to data source custodian’s website are provided in the Data Source section.

## 2022–2024 CDI Web Tool Refresh

The CDI web tool has received more than 1 million page views since it was first launched in January 2015. As part of the 2022–2024 CDI refresh, CDC also redesigned and enhanced the web tool to modernize the underlying application and improve the tool’s usability. Several key features and functions of the CDI web tool were maintained and enhanced.

### Indicator definitions and data source descriptions

The CDI web tool has consistently provided standardized definitions and detailed descriptions for each indicator. The detailed definitions include population, numerator, denominator, measure, time period of case definition, summary of background and importance, notes, data source, related objectives or recommendations, secondary CDI topic areas, and related references. This information helps users understand the meaning and context of the indicators.

With the CDI refresh, measure definitions were streamlined to ensure consistency across topic areas and indicators. Twenty-one new topic area landing pages were added to provide users a broad overview of the topic as well as links to where users can find more topic-specific estimates. A new data source section was added that provides information about the data sources, including full name and abbreviation, website, purpose, target population, frequency and mode of data collection, notes, treatment of missing data, data suppression, age-standardization process, and sponsor.

### Data visualizations

With the refresh, the CDI web tool maintained and enhanced the ability for users to create interactive charts, graphs, and maps. Users can create exhibits that present data in a visually appealing and easy-to-understand format. Users can select indicators, time periods, and population groups. The ability to stratify estimates by sex and race and ethnicity (when applicable) was retained from the previous version, and the refreshed CDI adds a stratification by age. These visualizations can help users identify patterns, trends over time, and disparities.

**Figure 1:**
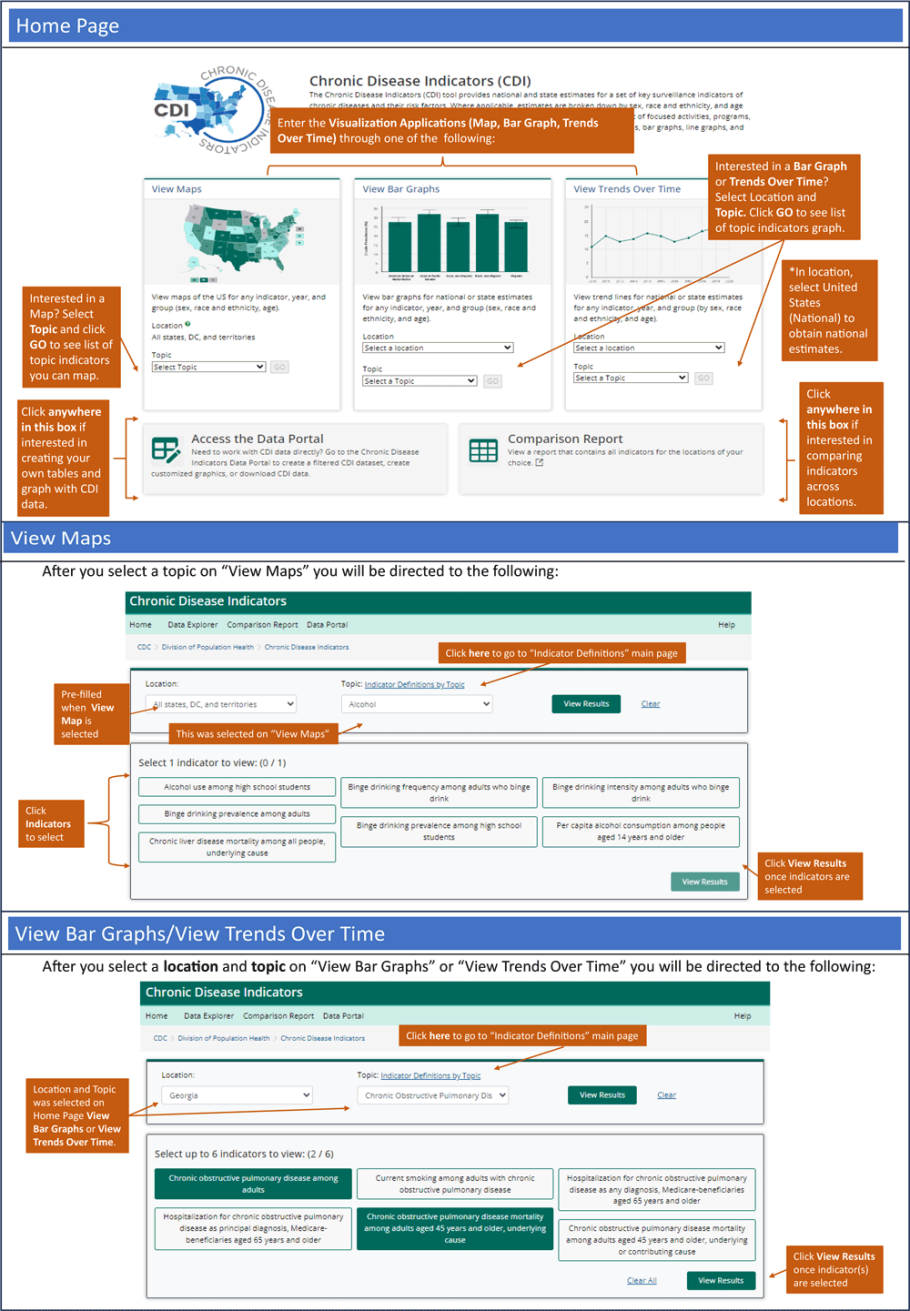
Annotated screenshot of the home page and entry points for applications in the Chronic Disease Indicators web tool (www.cdc.gov/cdi).

Entry into the data visualization section of the CDI web tool was enhanced and 3 entry points are provided: “view maps,” “view bar graphs,” and “view trends over time” (Figure 1).

**Figure 2:**
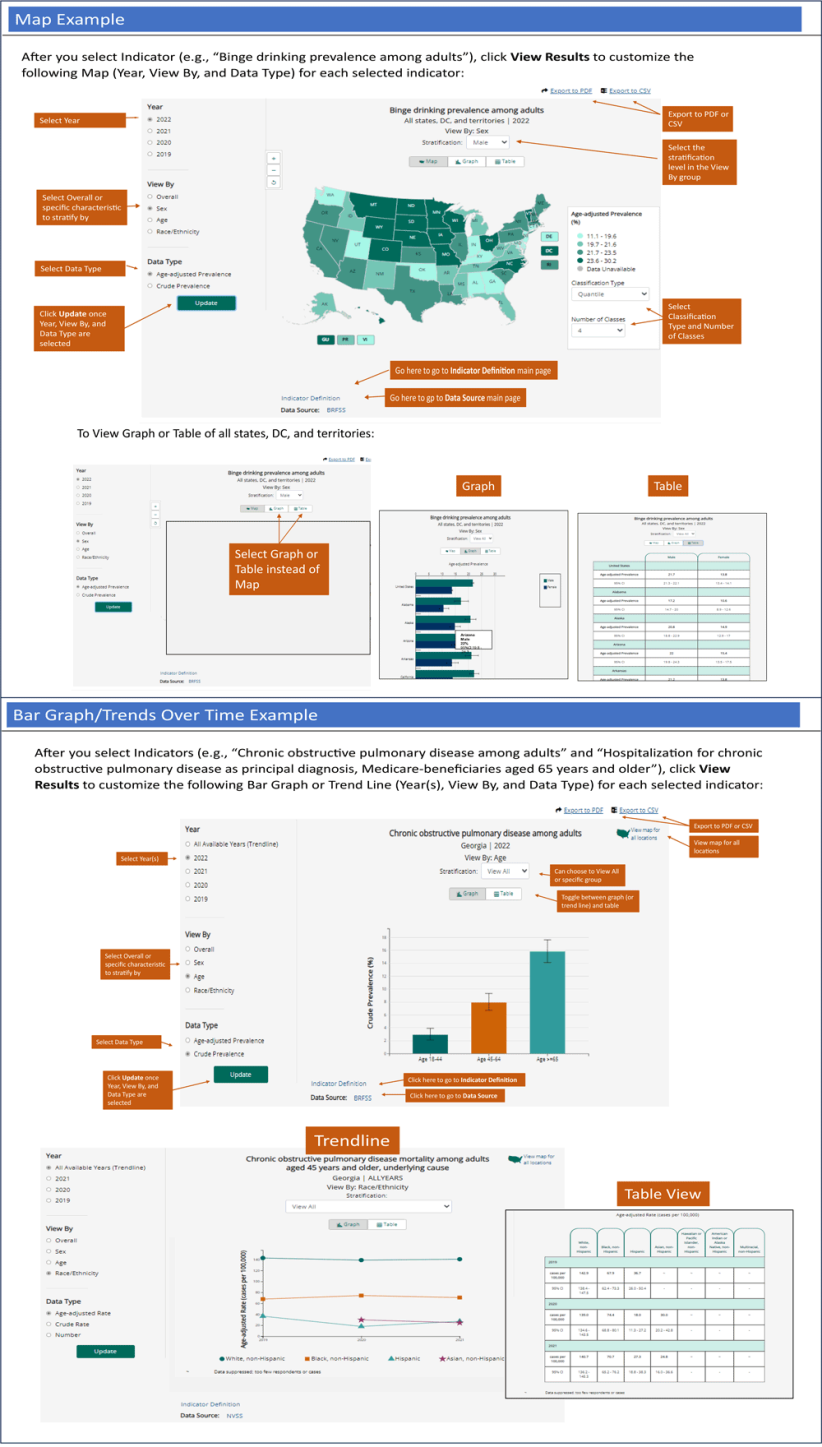
Annotated screenshot examples of the “view maps” and “view bar graphs/trends over time” features in the CDI Web tool (www.cdc.gov/cdi).

**“View maps.”** This entry point allows users to view maps of the US (all states, District of Columbia, and territories) for any indicator, year, and group (by sex, age, and race and ethnicity). To create the visualization shown in Figure 1 (middle section): 1) from the “view maps” box, select a topic area category from the drop-down menu (eg, alcohol) and click “go”; 2) select an indicator from the list (eg, binge drinking prevalence among adults); and 3) click “view results” to display the default map (overall estimates for most current year). To make changes (Figure 2, map example), users can select year, “view by,” and data type and select the classification type and number of classes. After selections are made, users click “update” to view the map choices selected. Last, when sex, age, or race and ethnicity is selected for the “view by” stratification, a map for a subgroup will be displayed (eg, male). Above the map, there is “view by” option for group level. When using the “view maps” option, only 1 indicator can be viewed at a time. Other visualizations provided in this section include a bar graph by state and a table with all state-level estimates for the indicator selected.

**“View bar graphs.”** This entry point allows users to view bar graphs for national or state-specific estimates for any indicator, year, and group (by sex, age, and race and ethnicity). To create the visualization shown in Figure 1 (“View Bar Graphs/View Trends Over Time”): 1) from the “view bar graphs” box, users select a location (eg, Georgia) and a topic area category from the drop-down menu (eg, chronic obstructive pulmonary disease); 2) select 1 or more indicators; and 3) click “view results” to display the default bar graph(s) (overall estimates for most current year) for all selected indicators. To make changes for each selected indicator (Figure 2, “Bar Graph/Trends Over Time Example”), users can select year, “view by,” and data type. After selections are made, users click “update” to view the graph with choices selected. Other visualizations provided in this section include an accompanying table with the estimates.

**“View Trends Over Time.”** This entry point allows users to view trend lines for national or state-specific estimates for any indicator, year, and group (by sex, age, and race and ethnicity). This entry point uses the same underlying application as View Bar Graphs; however, the default is to include all years of data available. Refer to the steps for View Bar Graphs.

### Comparison report

The comparison report allows users to view all estimates across states, enabling users to identify geographic disparities and variations in disease and risk factor prevalence (Figure 1). Users can select up to 4 locations, including estimates for the overall US and state-level estimates for each of the 50 states, the District of Columbia, and territories.

### Data portal

CDI data are published through an open data platform (https://data.cdc.gov/browse?category=Chronic+Disease+Indicators) that provides users an easy-to-use web-based interface to query and extract data based on their needs. Users can select indicators, geographic locations (national or state-specific), subgroups, and time periods to generate customized data sets and visualizations. Users can also find 21 smaller data sets with estimates for topic areas. With each CDI release, data sets are replaced to include any updated data. Data sets related to the previous version of the CDI (2015–2023) will not be updated but will remain available via the data portal.

## CDI Data in Action

State health departments, public health professionals, and policymakers can use the CDI web tool for diverse purposes. They can place a health problem in context by exploring their state’s prevalence of chronic conditions and associated behavioral risk factors. They can compare their data to national estimates and estimates in other states and monitor trends over time to guide decision-making for effective public health action. Decisions might include how to maximize limited resources by prioritizing and targeting health disparities for the most effective strategies to improve health outcomes while reducing disparities in diverse communities and populations.

At CDC, CDI provides estimates across multiple chronic diseases and their risk factors. In this role, CDI balances its presentation of indicators to effectively complement web tools hosted by other CDC programs, while also meeting the needs of smaller programs without dedicated data portals. For topics without dedicated data portals, select CDI data are often integrated into a topic’s website and links are provided to direct users to CDI for additional measures and estimates. For example, chronic obstructive pulmonary disease (COPD) is a health topic without a dedicated data portal. CDC’s COPD website (www.cdc.gov/copd) provides overall estimates of COPD prevalence and mortality and directs users to CDI for additional COPD measures and stratified estimates. For CDC programs with dedicated data portals, CDI incorporates key indicators for these topics and now provides users with links to additional data sources by topic area. This enhancement makes it easier for users to find estimates they need. For example, indicators related to nutrition, physical activity, breastfeeding, and obesity are provided on the Division of Nutrition, Physical Activity, and Obesity’s (DNPAO’s) Data, Trends, and Maps site (www.cdc.gov/nccdphp/dnpao/data-trends-maps) (13). While CDI includes 14 indicators for nutrition, physical activity, and weight status, DNPAO’s dedicated data portal includes almost 60 indicators that allow users a deeper dive into the topic.

External to CDC, state health departments and other organizations have used CDI data to provide evidence of need. The state health department in Nevada used estimates from the CDI in a presentation for a demonstration waiver for adults with diabetes (14). The Association of State and Territorial Dental Directors included the CDI as a data resource for state-level estimates for oral health indicators in their state surveillance reference guide for developing and implementing a robust state-level oral health surveillance system (15). State-level estimates on the adult population meeting the recommended amounts of physical activity provided by the CDI were included in the Bicycling & Walking in the United States 2018 Benchmarking Report (16). Finally, a publication from the official blog of the Association of Diabetes Care & Education Specialists used CDI estimates for the proportion of adults in Guam with diagnosed type 2 diabetes who have taken a diabetes self-management course to help describe the health care challenges facing US-affiliated Pacific Islands communities (17).

## Data Challenges

As with most sources of population health data, several data challenges must be considered. For example, sample size, data quality, and confidentiality considerations might limit the availability of data for some geographies and subgroups. Data reported in the CDI adhere to the criteria stipulated by the data providers, which are described in a new section on data sources. We suggest users be mindful of any major changes in how the indicator is assessed and exercise caution when comparing estimates for the same indicator over time. The “Notes to Users” section aims to provide such pertinent information (eg, changes to indicator definitions).

## Conclusion

CDI allows public health practitioners and others to visualize and download national and state chronic disease–related estimates across multiple topic areas and indicators. By refreshing CDI’s content and improving its ease-of-use, public health professionals, practitioners, and policymakers can better use these data and tools to guide health planning and actions to improve health.
